# Embolization of chest wall varicosity for Mondor disease manifesting as pulmonary embolism

**DOI:** 10.1016/j.jvscit.2025.101907

**Published:** 2025-07-05

**Authors:** Fatima Mustansir, Faiz Gani, Nathan M. Droz, Robert W. Thompson, Zachary J. Wanken

**Affiliations:** a Division of Vascular Surgery, Department of Surgery, WashU Medicine, St Louis, MO; b Department of Surgery, WashU Medicine, St Louis, MO; c Division of Vascular Surgery, Department of Surgery, University of Utah, Salt Lake City, UT

**Keywords:** Mondor, Embolism, Vascular plug

## Abstract

Mondor disease is a rare disease consisting of superficial thrombophlebitis of veins of the anterior thoracic wall, and less often of the groin, axilla, and abdomen. It most commonly presents as a cord-like induration on the chest wall. The etiology is unclear although it has been associated with breast cancer, local trauma, and hypercoagulable states. It is typically a benign and self-limiting condition, with pain being the most common complaint. We present the case of an otherwise healthy young male patient who presented with a chest wall thrombosed varicosity of an axillary vein tributary, which was a nidus for a subsequent pulmonary embolism. He was treated with a vascular plug to occlude the varicosity and prevent further pulmonary emboli.

## Case report

**Fig 1:**
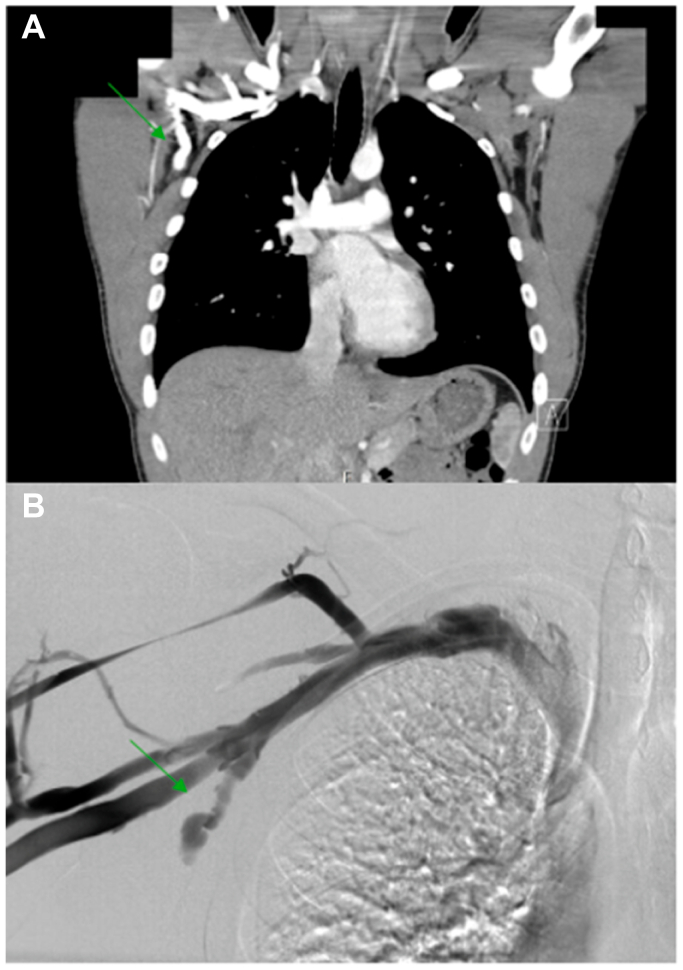
Preoperative **(A)** computed tomography (CT) angiogram, coronal view, and **(B)** venogram demonstrating the chest wall varix (*green arrows*).

A 21-year-old man, who consented to publication of his clinical course and had no significant past medical history, was referred to the Vascular Surgery Thoracic Outlet Syndrome center for further evaluation of a right axillary/chest wall mass in the setting of a newly diagnosed pulmonary embolism (PE). The patient described feeling a firm nontender lump in his right axilla running along his chest wall, which he first noticed approximately 6 months ago. He denied associated arm swelling, pain, numbness, or tingling; chest pain; dyspnea; weight loss; or antecedent trauma. He is a college student and naval cadet, who was undertaking naval training exercises at the time. He had been evaluated previously by multiple physicians for the same problem, including his primary care provider, who initiated workup with a chest computed tomography (CT) scan, which revealed bilateral segmental and subsegmental PE along with a long tubular structure adjacent to the right axillary vein most consistent with axillary venous thrombosis (Fig 1, *A*). The patient had a transthoracic echocardiogram performed shortly after this CT scan was done, which noted a normal ejection fraction, with a dilated right ventricle and borderline dilated right atrium. The right ventricular systolic pressure was unable to be measured. He has not had additional echocardiograms since then. Upper and lower extremity venous duplex ultrasound studies were obtained, which also showed a nonocclusive thrombus in the right axillary vein. He was placed on oral anticoagulation with rivaroxaban. He underwent a right upper extremity venogram (Fig 1, *B*) at an outside facility before presenting to our hospital, which demonstrated a patent right axillary-subclavian vein without thrombus. However, a nonthrombosed varicosity arising from a branch of the axillary vein (possibly the lateral thoracic vein) was observed. A follow-up CT angiogram showed interval resolution of his PE. On physical examination, he had palpable right radial and ulnar pulses, normal motor and sensory function, and absence of positive findings after provocative maneuvers for thoracic outlet syndrome. An approximately 0.5 × 2 cm firm, nontender, palpable, cord-like mass was palpated in the right axilla, running vertically along the right upper chest wall. There was no erythema, warmth, palpable thrill, or surrounding distended veins.

His presentation was not consistent with venous thoracic outlet syndrome or an arteriovenous malformation. It was recommended that he undergo percutaneous venogram and embolization of the chest wall varicosity, which was felt to be a form of Mondor disease, to prevent further embolization. This definitive management would allow him to transition off anticoagulation so he could resume his physical activities that were an essential part of his job.

**Fig 2:**
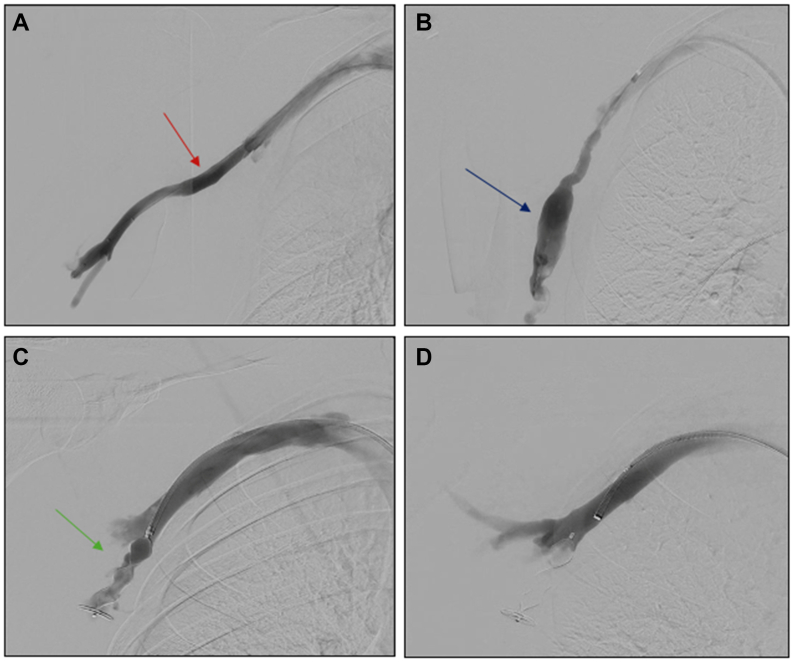
Diagnostic venogram demonstrating **(A)** a patent axillary vein (*red arrow*) and **(B)** a venous varicosity off the axillary vein, and likely lateral thoracic vein (*blue arrow*). The neck of varix the measures 10 mm. **(C)** Vascular plug placed to successfully occlude the varicosity (*green arrow*). **(D)** Fully deployed plug with completion venogram showing absence of flow into the varix beyond the plug.

He was taken to the operating room for a right upper extremity venogram via common femoral vein access. The initial venogram showed a widely patent axillary vein (Fig 2, *B*). Selective catheterization of the varix was performed using an appropriate wire and sheath combination. A 7 F treatment sheath was advanced into the varix over a stiff 0.035” guidewire. The culprit varicosity was identified arising from a branch of the axillary vein, likely the lateral thoracic vein (Fig 2, *B*), which was delineated with venography. A 16-mm Amplatzer II plug (Abbott Cardiovascular, St Paul, MN), which was appropriately oversized relative to the neck of the varix (which measured around 10 mm on preoperative CT imaging), was chosen. It was delivered into the varix and deployed (Fig 2, *C* and *D*). A postdeployment venogram demonstrated excellent positioning within the varicosity without impeding flow through the main axillary vein.

**Fig 3:**
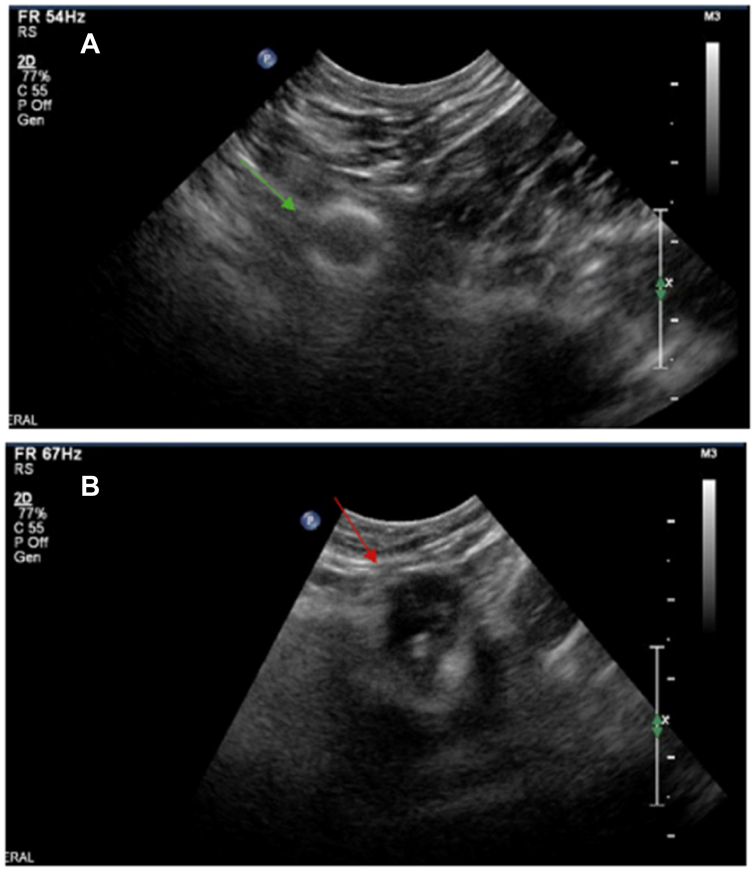
Postoperative duplex ultrasound image showing **(A)** stable placement of the vascular plug within the chest wall varix (*green arrow*) and **(B)** thrombosed, noncompressible chest wall varix (*red arrow*).

He was seen in the clinic on two occasions postoperatively, where he was found to be recovering well with patent deep veins on the upper extremity venous duplex ultrasound examination. He was continued on oral anticoagulation for 3 months after his procedure. A repeat duplex ultrasound examination (Fig 3, *A* and *B*) after he was off anticoagulation demonstrated patent deep veins and involution of the embolized varix. At that time, his ongoing risk for deep venous thrombosis (DVT)/PE was determined to be low, given successful treatment of the embolic source.

## Discussion

Mondor disease was described by French surgeon Dr Henri Mondor in 1939.^1^ It is a type of superficial thrombophlebitis most commonly involving the anterior chest wall, followed by the groin, axilla, or abdomen via thoraco-epigastric, superior epigastric, and lateral thoracic veins.^2^, ^3^, ^4^ It generally manifests as an indurated palpable cord on the chest wall or breast that may be painful, especially while stretching or moving the upper extremity. It occurs in a preponderance of middle-aged female patients. It is usually idiopathic, but has been linked to factors that promote thrombosis such as trauma, surgery, infections, venous compression, and breast cancer.^3^^,^^5^ Its pathogenesis involves an acute phlebitis stage, with progression to thrombophlebitis and sclerosis with either recanalization or obliteration of the vein.^6^ Ultrasound examination is the diagnostic modality of choice for Mondor disease. In patients who do not demonstrate resolution of Mondor disease within a few weeks, their symptoms can be managed with warm compresses and nonsteroidal anti-inflammatory drugs. Systemic anticoagulation is not indicated routinely for superficial thrombophlebitis, especially because recurrence is rare.^7^ However, systemic anticoagulation has been used in isolated instances with success, although it is difficult to determine whether anticoagulation plays a significant role in the resolution of the disease process.^8^

In this case, surgical management of the patient's chest wall varicosity was necessary so that he could discontinue anticoagulation and resume his physical activities safely. We opted for a minimally invasive endovascular approach for this young muscular patient to avoid the morbidity of a large incision to reach the varix, which was approximately 3 cm below the skin level, and the axillary vein and deep venous confluence. Advances in vascular plug technology have rendered these devices safe; they are able to be deployed precisely with a low risk of migration, target vessel recanalization, and erosion.^9^^,^^10^

The reported incidence of Mondor disease is <1%, in part because the disease is under-reported by patients because of its self-limiting nature.^3^^,^^7^^,^^11^ Existing reports of Mondor disease highlight its cutaneous manifestations, with the aim of improving its recognition because it may herald underlying breast cancer or a systemic disorder that predisposes to hypercoagulability.^7^^,^^12^

Although the association of superficial venous thrombosis of the lower extremity (which mostly involves the great saphenous vein) with more clinically significant pathologies such as concurrent DVT and PE 13, 14, 15 has been shown in several studies, DVT/PE are less common with upper extremity superficial venous thrombosis (which mostly involves the cephalic vein) and have not been investigated as thoroughly.^16^ A French study of 160 patients with upper extremity DVT and superficial venous thrombosis showed that only 2.5% of patients (n = 4) had PE and that superficial venous thrombosis was frequently associated with a cord-like induration.^17^ The relationship between lower extremity superficial venous thrombosis and DVT/PE has led to studies examining the effectiveness of a course of anticoagulation for lower extremity superficial venous thrombosis and helped to change practice patterns.^18^

There are only a handful of case reports of PE associated with upper extremity superficial venous thrombosis involving the basilic or cephalic veins.^19^, ^20^, ^21^ To our knowledge, this case of upper extremity superficial venous thrombosis in a varicosity in proximity to the axillary vein causing PE and, therefore, as a direct result of Mondor disease.

## Conclusions

PE in the setting of upper extremity superficial venous thrombosis is a rare occurrence. However, an appropriate index of suspicion should be maintained in such cases to provide appropriate treatment. In this case of a young patient with Mondor disease, endovascular intervention to occlude a varicosity of the lateral thoracic vein was necessary to provide complete resolution of the disease process.

## Funding

None.

## Disclosures

None.

## References

[1] Kyle R.A., Shampo M.A. (1986). Henri Mondor: biographer and surgeon. Mayo Clin Proc.

[2] Alvarez-Garrido H., Garrido-Ríos A.A., Sanz-Muñoz C., Miranda-Romero A. (2009). Mondor’s disease. Clin Exp Dermatol.

[3] Renshaw L., Dixon J.M., Anderson J., Turnbull A.K. (2022). Mondor’s disease of the breast: a cutaneous thromboembolic manifestation of Covid-19?. Breast.

[4] Vijayan P., Pang F.T. (2023). Mondor’s disease of the breast in Asia: a forgotten diagnosis for the front-line clinicians—a case report and literature review. Egypt J Radiol Nucl Med.

[5] Amano M., Shimizu T. (2018). Mondor’s disease: a review of the literature. Intern Med.

[6] Roscher A.A., Weinstein E. (1980). The clinico-pathological spectrum of Mondor’s disease: an important surgical entity. Int Surg.

[7] Salemis N.S., Merkouris S., Kimpouri K. (2011). Mondor’s disease of the breast. A retrospective review. Breast Dis.

[8] Belleflamme M., Penaloza A., Thoma M., Hainaut P., Thys F. (2012). Mondor disease: a case report in ED. Am J Emerg Med.

[9] Lopera J.E. (2015). The Amplatzer vascular plug: review of evolution and current applications. Semin Intervent Radiol.

[10] Belachsen O., Sargent J., Koffas H., Schneider M., Wagner T. (2022). The use of Amplatzer vascular plug II in 32 consecutive dogs for transvenous occlusion of patent ductus arteriosus. J Vet Cardiol.

[11] Okumura T., Ohhira M., Nozu T. (2012). High rate of smoking in female patients with Mondor’s disease in an outpatient clinic in Japan. Int J Gen Med.

[12] Crisan D., Badea R., Crisan M. (2014). Thrombophlebitis of the lateral chest wall (Mondor’s disease). Indian J Dermatol Venereol Leprol.

[13] Decousus H., Quéré I., Presles E. (2010). Superficial venous thrombosis and venous thromboembolism: a large, prospective epidemiologic study. Ann Intern Med.

[14] van Weert H., Dolan G., Wichers I., de Vries C., ter Riet G., Buller H. (2006). Spontaneous superficial venous thrombophlebitis: does it increase risk for thromboembolism? A historic follow-up study in primary care. J Fam Pract.

[15] Di Minno M.N.D., Ambrosino P., Ambrosini F., Tremoli E., Di Minno G., Dentali F. (2016). Prevalence of deep vein thrombosis and pulmonary embolism in patients with superficial vein thrombosis: a systematic review and meta-analysis. J Thromb Haemost.

[16] Bell L.N., Berg R.L., Schmelzer J.R. (2017). Thromboembolic complications following a first isolated episode of superficial vein thrombosis: a cross-sectional retrospective study. J Thromb Thrombolysis.

[17] Drouin L., Pistorius M.-A., Lafforgue A. (2019). [Upper-extremity venous thrombosis: a retrospective study about 160 cases]. Rev Med Interne.

[18] Decousus H., Prandoni P., Mismetti P. (2010). Fondaparinux for the treatment of superficial-vein thrombosis in the legs. N Engl J Med.

[19] Guertler A., Haas C., Sattler E. (2020). [Bilateral pulmonary embolism after superficial thrombophlebitis of the upper extremity]. Hautarzt.

[20] Sassu G.P., Chisholm C.D., Howell J.M., Huang E. (1990). A rare etiology for pulmonary embolism: basilic vein thrombosis. J Emerg Med.

[21] Cascella M., Viscardi D., Bifulco F., Cuomo A. (2016). Postoperative massive pulmonary embolism due to superficial vein thrombosis of the upper limb. J Clin Med Res.

